# Sydney epilepsy incidence study to measure illness consequences: the SESIMIC observational epilepsy study protocol

**DOI:** 10.1186/1471-2377-11-3

**Published:** 2011-01-09

**Authors:** Maree L Hackett, Nicholas S Glozier, Alexandra L Martiniuk, Stephen Jan, Craig S Anderson

**Affiliations:** 1 Neurological and Mental Health Division, The George Institute for Global Health, The University of Sydney, Sydney, Australia; 2 Psychological Medicine, The University of Sydney, Sydney, Australia; 3 Injury Division, The George Institute for Gloabal Health, The University of Sydney, Sydney, Australia; 4 Renal Division, The George Institute for Global Health, The University of Sydney, Sydney, Australia

## Abstract

**Background:**

Epilepsy affects an estimated 50 million people and accounts for approximately 1% of days lost to ill health globally, making it one of the most common, serious neurological disorders. While there are abundant global data on epilepsy incidence, prevalence and treatment, there is a paucity of Australian incidence data. There is also a general lack of information on the psychosocial impact and socioeconomic consequences of a new diagnosis of epilepsy on an individual, their family, household, and community which are often specific to the health and social system of each country.

**Methods/Design:**

The Sydney Epilepsy Incidence Study to Measure Illness Consequences (SEISMIC) is an Australian population-based epilepsy incidence and outcome study that will recruit every newly diagnosed case of epilepsy in the Sydney South West Area Health Service to an epilepsy register. Multiple and overlapping sources of notification will be used to identify all new cases of epilepsy over a 24 month period in the Eastern Zone of the Sydney South West Area Health Service (SSWAHS) and follow up will occur over 12 months. SEISMIC will use the International League Against Epilepsy (ILAE) definitions and classifications for epidemiologic studies of epilepsy. The study will examine outcomes including mood, quality of life, employment, education performance, driving status, marital and social problems, medication use, health care usage, costs and stigma.

**Discussion:**

This study is designed to examine how clinical, psychological factors, socioeconomic circumstances, and healthcare delivery influence the experience of epilepsy for individuals and families allowing better targeting of specific services and informing policy makers and practitioners. In addition, the study will provide the basis for a longitudinal population-based cohort study and potentially inform qualitative sub-studies and randomised controlled trials of intervention strategies. The study has been registered on the Australia New Zealand Clinical Trial Registration database with ANZCTRN12609000059268.

## Background

Currently there is very limited evidence about the burden of epilepsy in the Australian population. The best available estimates based on a cross sectional survey in Tasmania and a study conducted 30 years ago suggest that it is prevalent in between 6 and 7.5 per 1000 people [1,2]. This equates to approximately 150,000 people in Australia presently living with this condition making it one of the 50 leading causes of disease burden and injury in the country [3]. Globally about 50 million people are affected by epilepsy, which accounts for about 1% of days lost to ill health globally [4]. The average incidence across developed countries has been estimated at 43.4/100,000 [5] which would suggest that over 8,800 Australians develop the condition every year. Despite the potential magnitude of the burden of epilepsy, population-based Australian data on the natural history of epilepsy are limited, and strategies to reduce the considerable psychosocial and economic impact of epilepsy are lacking, or poorly understood.

Epilepsy is a chronic neurological disorder with the fundamental characteristic of recurrent, intermittent seizures unprovoked by any acute medical condition or transient brain disorder. A diagnosis of epilepsy is made in those more than 44 weeks post conception, following a second unprovoked seizure occurring at least 24 hours after the first. However "epilepsy" incidence studies have used varying definitions, often including people with single seizures.

Age-specific incidence of epilepsy is bimodal: it is high in those aged over 60 years and highest in children and adolescents, with a slight predominance in males [5,6]. However, it is commonly thought that incidence rates are underestimated in the elderly due to incomplete case ascertainment in studies. Three recent studies have demonstrated a higher incidence in lower income groups in the United Kingdom, New York and Iceland [7-9] and incidence appears to be higher in developing countries [5].

Epilepsy is associated with increased mortality [10], including increased risk of sudden unexpected death (SUDEP) [11], and morbidity: this may be physical (e.g. fractures, scalding, bruising) occurring directly as result of seizures, cognitive (e.g. cognitive delay, speech or language difficulties, learning disabilities), behavioural (e.g. aggression) [12,13], or psychosocial (e.g. depression, anxiety, stigma).

### Seizure Treatment

The goal of initial treatment is to achieve seizure control by preventing seizure recurrence and maintaining optimal physical, cognitive, behavioural and emotional function. Approximately 60-70% of people with new onset epilepsy become, and remain, seizure free with appropriate medication [14]. In many of these cases drug treatment can be withdrawn after seizure remission, although the risk of relapse remains high [15]. However, about one third of incident cases have recurrent seizures [6]. The longer term influence of antiepileptic drugs is unknown [16]. Despite the magnitude of the problem, and the effectiveness and relatively low cost of treatment, many patients may not receive effective treatment through lack of adherence to medication, reduced availability of diagnosis and drugs e.g. living in a rural area or inability of the individual or health system to pay for treatment, such as in a low income country [17]. Further, there may be delays in seeking help due to ignorance, fear, stigma [18], illiteracy and cultural attitudes towards treatment (e.g. attributing epilepsy to possession).

### The psychosocial impact of epilepsy

The social sequelae of epilepsy are far reaching and potentially include the loss of a driving licence, lack of access to or change in employment or education circumstances, marital and social problems, taking regular medication and dealing with the responses of others to the diagnosis including perceiving and experiencing stigma and discrimination [18]. Reduced social participation, physical inactivity, and higher healthcare usage are also more common in people with epilepsy [19]. As a group, people with epilepsy have poorer psychosocial functioning than those without epilepsy [20].

Psychological comorbidities such as anxiety, depression [21-23] and sleep disturbance [24] are common in people with epilepsy, and increase the risk of suicide [25]. Lifetime prevalence estimates of major depressive disorder are as high as 17.4% (95% CI 10-24.9), mood disorders 24.4% (16.0-32.8) and anxiety disorders 22.8% (14.8-30.9) in people with epilepsy, who were more than twice as likely than people without epilepsy to report such disorders [26]. Problems in the areas of attention, cognition and socialisation are also apparent in children with epilepsy [27].

Psychosocial sequelae have a greater impact on health-related quality of life (HRQoL) than short-term seizure control [28,29]. Veterans with epilepsy with a comorbid psychiatric diagnosis (depression, anxiety, substance abuse or post traumatic stress disorder) had HRQoL scores 24% lower on all eight domains the SF-36 than veterans with epilepsy alone [30]. People with epilepsy and depression are more likely to report having seizures than those without depression [31] and seizure activity explains much of the variation in psychosocial functioning [22]. Whilst more frequent seizures are associated with impaired HRQoL, and even infrequent seizures (less than one per year) impair HRQoL in older adults, stigma is found to be the strongest predictor of reduced HRQoL [20,29,32]. Despite psychosocial impairment accounting for a large proportion of the negative outcomes experienced by people with epilepsy, there has been little investigation into the natural history of these psychosocial problems [33]. While the data are limited, there may be some association between marital (unmarried/separated/divorced) [31,34] and employment (unemployed) [31,34] status, increasing number of medical problems [31], lower education, income [35], activity, social support and increased stigma [34] and the risk of depression.

The development of interventions in this area lags behind pathophysiological and pharmacological research. It has been postulated that stigma may be moderated by individual resilience-based factors such as self esteem and social support which, if confirmed, offer promising areas for intervention [36]. However, there is also little information on the nature of social support required by patients, or the impact of epilepsy on carers, the family or the household.

### The economic impact of epilepsy

There are limited data on the economic impact of epilepsy on the individual, their family or the community. Despite calls from the ILAE in 1997 for cost-utility analyses to measure psychosocial and other subjective patient-reported outcomes [37], economic data collected to date are limited to socioeconomic disparities in incidence, epilepsy-related health service costs and gaps in delivery of appropriate treatment [8,17,38]. It is possible that socioeconomic disparities in incidence (increased incidence with more socioeconomic deprivation) may be a result of confounding [8]. The highest expenditure for the household of individuals with epilepsy appears to occur in the first year after diagnosis which reflects higher use of specialist services over that period [38]. However, when considering costs to a country, a large proportion of the burden comes from indirect costs such as unemployment and early mortality [39]. There is an urgent need for reliable information on the direct and indirect costs of the condition and for a better understanding of the factors that are associated with adverse economic outcomes for patients, their families and the community.

### Australian data

A high quality longitudinal, prospectively recruited population-based sample of Australians with incident epilepsy is lacking. The limited Australian epidemiological epilepsy data are predominantly from cross-sectional surveys of people currently accessing health care for epilepsy, in addition to some early studies of Sydney general practitioners' knowledge of, management of and attitudes towards epilepsy [40]. An early Sydney study using '3 or more seizures verified by a doctor' to define epilepsy found a prevalence of 7.5 per 1,000, with remission (30% off medication) and medication usage (70%) as the primary endpoints [1]. This prevalence estimate was adjusted to 1 in 50 in a subsequent analysis [41]. A retrospective study on SUDEP in Victoria [42] which led to a prospective case-cohort coronial deaths study of SUDEP found that young people with epilepsy are more likely to die in their sleep than their counterparts [43]. More recently a survey of Tasmanian residents on the Australian National prescription database who had been prescribed an antiepileptic drug for epilepsy or seizures was conducted. This study showed that one quarter of participants reported very high levels of psychological distress from anxiety and mood disorders, with this group also having higher usage of healthcare services [44].

### Scientific rationale for a study of newly diagnosed epilepsy in Australia

The gold standard study design to determine the true impact of epilepsy is a large-scale, longitudinal, prospective, population-based incidence and outcome study which allows observation of the natural history of all aspects of epilepsy across the age and severity spectrum. Such a design will enable identification of potentially modifiable determinants of outcome including clinical, psychological, social and economic factors that reduce vulnerability or enhance resilience to the psychosocial and socioeconomic consequences of epilepsy in Australia.

## Methods/Design

### Design and Overview

The Sydney Epilepsy Incidence Study to Measure Illness Consequences (SEISMIC) is a prospective, population-based epilepsy incidence and outcome study with study registration number ACTRN12609000059268. The primary aim of SEISMIC is to identify modifiable factors that enhance resilience and reduce vulnerability to the socioeconomic impact of epilepsy in an Australian population. We hypothesise that a) psychological factors (depressive and anxiety symptom burden, and coping) are associated with socioeconomic outcomes (school performance, work absence, family impact and economic hardship), and b) social and economic deprivation, and service access factors are associated with variation in disability and clinical outcomes from epilepsy.

### Study Population

#### Inclusion criteria

The population to be surveyed and monitored includes people more than 44 weeks post conception (i.e. excluding neonates) fulfilling all of the following criteria:

• New diagnosis of epilepsy, and

• Residing within the SSWAHS Eastern Zone geographical boundaries for the past month

• They or their "responsible person" (relative/friend) are able to provide written informed consent. Participants with a severe language disorder or cognitive impairment (as indicated by their clinician) are eligible to take part in the study providing their relative/friend is able to provide written informed consent and complete the assessments on the participant's behalf), and

• Consent is given to contact the GP if necessary in the case of a severe psychiatric disorder warranting treatment.

#### Exclusion criteria

• Seizures due to acute cerebral insult or reversible metabolic courses.

### Population Base

The population to be surveyed and monitored includes those who are usually resident in the Eastern Zone of the SSWAHS http://www.sswahs.nsw.gov.au. In 2006 the estimated resident population of the SSWAHS was 510,000 people, and comprised 7 local government areas. Children under the age of 15 constitute 16% of the population; people aged 65 years and over make up about 12% and indigenous people comprise 1% of the population. This area is characterized by its multicultural character, with 39% speaking a language other than English at home. Furthermore this population has some of the poorest communities in the State, characterized by a large number of recent migrants, significantly higher unemployment and a high proportion of families dependent on welfare. Overall 40% of the area's population live in rented accommodation. The following hospitals or health-care centres are either in the Eastern Zone of the SSWAHS or treat people with epilepsy who reside within the catchment area: Bankstown Hospital, Brain and Mind Research Institute, Concord Repatriation Hospital, Prince of Wales Hospital, Royal Prince Alfred Hospital, St Vincent's Hospital, Sydney Children's Hospital at Randwick, Westmead including Westmead Children's Hospital. The rooms of private epileptologists, neurologists and paediatricians will also operate as recruiting sites.

### Case ascertainment and consent

The lead clinician at each public or private site will act as the "principal investigator" (PI). The PI will be required to consent potentially eligible cases, or their parent (for potential cases aged 18 years or under), or guardian (for potential cases unable to consent themselves) of people with new diagnoses of epilepsy into the study. Each PI will maintain a log of every potentially eligible patient who is approached to participate in the study. The logs contain the initials and date of birth of the eligible patients, the date they were screened for participation in the study, whether or not consent to participate was obtained and if not, the reason for their non-participation. Participants consent to: 1) the abstraction of information about their epilepsy from medical records; 2) interviews at baseline (as soon as possible after diagnosis), four months and one year (primary endpoint), and; 3) for the participant's general practitioner (GP) to be contacted by research staff if necessary. All PIs or their research staff are contacted weekly to track screening and recruitment.

To ensure identification of all people with a new diagnosis of epilepsy in the SSWAHS who are not seen in a public hospital, regular liaison and collaboration with General Practitioners (GP), paediatricians and private neurologists in the area is essential. Various methods will be used to initiate and maintain a high level of awareness of the study in the community, including 6 monthly contacts to enquire about potentially eligible participants, and provision of pamphlets, magnets, posters and training for clinicians and allied health professionals.

In addition PIs may consent participants with their first unprovoked seizure, who do not currently meet the criteria for a diagnosis of epilepsy, into the SEISMIC First Seizure substudy. These people will be asked to allow 6 monthly contact from the research team to ascertain if there has been any clinical changes that would warrant their subsequent inclusion as a case of new onset epilepsy over the course of the study's recruitment.

### Assessments and data collection

The detailed schedule for data collection is shown in Table 1. Researchers will travel to the recruitment site of consented participants and abstract the following data from consented participants' medical notes: initials, date of birth, sex, contact information, source of notification (hospital, private site, general practitioner etc) and clinical information. Data are entered onto electronic case report forms (eCRFs) and uploaded to a secure centralised database.

**Table 1:** Schedule of assessments in the SESIMIC study

Participant Assessment^a^	Baseline^b^	4 month	12 month
Initials	X		

Date of birth	X		

Sex	X		

Inclusion criteria	X		

Contact information	X		

***Interview Characteristics***			

Method of assessment	X	X	X

Participant or proxy	X	X	X

Alive at scheduled time of assessment	X	X	X

Telephone Interview for Cognitive Status (TICS) [63]	X	X	X

***Seizure characteristics ***[45]			

Consciousness	X		

Onset	X		

Frequency	X	X	X

Phenomena	X	X	X

Precipitants	X		

Family history	X		

Epilepsy-related medical history	X		

New medical problems		X	X

Modified Cumulative Illness Rating Scale [66]	X	X	X

Birth History	X		

Medication	X	X	X

Investigations (EEG, CT, MRI etc)	X	X	X

Accidents/falls/injuries		X	X

Access to services		X	X

Final diagnosis		X	

Developmental milestones		X^c^	X^c^

Cognitive assessment (optional)		X	X

***Demographic characteristics***			

Born in Australia	X		

Years lived in Australia	X		

Language other than English spoken at home	X		

Marital status	X	X	X

Number of financially dependent children	X		

Social/financial living status	X	X	X

***Medical History***			

Psychotropic medications	X	X	X

Brief Illness Perceptions Questionnaire [48]	X	X	X

WHO Disability Assessment Schedule [67]	X^a^	X^a^	X^a^

Strengths and Difficulties Questionnaire [50]	X^c^	X^c^	X^c^

Alcohol consumption: Audit Alcohol Use Disorders Identification Test (AUDIT-C) [51]	X^a^	X^a^	X^a^

***Education and Employment***			

Highest qualification	X	X	X

Current student	X	X	X

Absences from education	X	X	X

Lifetime occupation	X	X	X

Current occupation	X	X	X

Job content questionnaire [53]	X^a^	X^a^	X^a^

***Financial Situation***			

Household benefits	X	X	X

Main source of income	X	X	X

Insurance	X	X	X

Economic hardship	X	X	X

Income	X	X	X

***Transport***			

Vehicles driven and frequency	X^a^	X^a^	X^a^

Transport to work/education	X^a^	X^a^	X^a^

Drive for work	X^a^	X^a^	X^a^

***Childcare***			

Location	X^c^	X^c^	

Duration	X^c^	X^c^	

Absence from childcare	X^c^	X^c^	

***Support***			

Confiding relationship	X	X	X

Family adaptation, partnership, growth affection, resolve questionnaire [55]	X	X	X

Inventory of Family Protective Factors [56]	X	X	X

Social media use	X	X	X

***Mood***			

Hospital Anxiety and Depression Scale [57]	X^a^	X^a^	X^a^

General Health Questionnaire	X^c^	X^c^	X^c^

***Stigma***			

Modified HIV Stigma Scale [59]	X	X	X

Disclosure of epilepsy diagnosis	X	X	X

***Sleep***			

Pittsburgh Sleep Quality Index [60]	X	X	X

***Health related quality of life***			

EuroQol [47]	X^a^	X^a^	X^a^

Quality of Life in Epilepsy for Adolescents [61]	X^b^	X^b^	X^b^

Longitudinal Study of Australian Children [62]	X^d^	X^d^	X^d^

^a ^Adult only (> 18 years of age)

^b ^Adolescent only (11-18 years of age)

^c ^Child only (< 18 years of age)

^d ^Young child (5-10 years of age)

#### Baseline interview

This will be conducted through a structured face to face interview by a trained researcher at a place of the participant's or their family's convenience. Participants complete a clinical and an age-specific psychosocial assessment.

*The clinical assessment for all participants, or their parent, proxy, carer or guardian *includes a clinical and modified-Austin interview [45]. This questionnaire collects information on epilepsy onset and prodromal characteristics, seizure type classification and syndromal classification, complete description of seizure: type, duration, and frequency; other ictal phenomena such as falls; and duration of post-ictal recovery, any symptom side effects if prescribed antiepileptic drugs, details of neurological investigation results (e.g.: EEG, MRI etc) and medical factors potentially associated with morbidity including comorbidity, family history and potential aetiological factors e.g. head injury, birth asphyxia.

Following completion of the clinical assessment, participants or their proxy complete either the adult/guardian or the parent/carer of a child assessment as appropriate.

*The adult psychosocial interview with participants who are 18 years of age or older *consists of detailed demographic, psychosocial and socioeconomic questions including: duration of residence in Australia, physical and social living situation, medical history, general wellbeing, education and employment, household financial circumstances, transport, social support, mood, stigma and sleep. At the end of the interview, all participants are given the opportunity to provide any other information about their experience or circumstances that has not been covered during the interview.

*The parent or carer psychosocial interview for participants who are between 0 and 18 years of age *consists of the same information as above with the addition of questions about childcare. Adolescents (between 11 and 18 years of age) and children (between 5 and 10 years of age) also complete age-specific quality of life questionnaires. At the end of the interview, all participants are given the opportunity to provide any other information about their experience or circumstances that has not been covered during the interview.

#### 4 month interview

Participants complete a clinical and an age-specific psychosocial assessment covering the four month period since their diagnosis. *The clinical assessment for all participants *consists of a review of seizure frequency since last interview, clinical investigations, medications, general health, developmental milestones and an optional cognitive assessment if deemed necessary by the interviewer. *The adult, parent or carer interviews *are as outlined previously.

#### 12 month interview

Participants complete a clinical and an age-specific psychosocial assessment covering the previous 8 month period. *The clinical assessment for all participants *is as outlined at the four month assessment with a reassessment of the diagnosis of epilepsy or epilepsy syndrome. *The adult, parent or carer interviews *are as outlined previously.

#### Specific questionnaires used include

The *EuroQol *(EQ-5D) [46,47] is a measure of quality of life that provides a simple descriptive profile and a single value for health status graded on a scale from -0.59 to 1.00. Lower scores indicate poorer quality of life. A score of 0 represents no quality of life and scores less than 0 represent states perceived by the respondent to be worse than death.

The *Brief Illness Perception Questionnaire *(BIPQ) [48] is a valid and reliable measure of patients' cognitive and emotional representations of their illness across a variety of illness groups. The BIPQ assesses consequences, timeline, personal control, treatment control, identity, coherence, concern, emotional response, and causes of illness. Items are rated using a 0 (not at all) to 10 (severely affects my life) response scale.

Psychosocial disability is assessed using the *12-item World Health Organisation Disability (WHO) Assessment Schedule *(WHO-DAS II) [49]. The 12-item WHO-DAS II assesses the magnitude of disability over the previous 30 days using a five point scale from none to extreme/cannot do. Total scores range from 0-100 with higher scores indicating greater disability.

The *Strengths and Difficulties Questionnaire *(SDQ) [50] is a brief behavioural screening questionnaire for parents of 4 to 16 year olds which screens for child psychiatric disorders. In addition to assessing common emotional and behavioural difficulties, it also asks if the parent/carer/proxy thinks that the child has problems in these areas and if there is associated distress and social impairment. The SDQ consists of 5 domains with 5 items per domain with responses ranging from 0 (not true) to 2 (certainly true) with higher scores indicating more problems.

The A*lcohol Use Disorders Identification Test *(AUDIT-C) [51] uses three questions to collect information on 'at-risk' alcohol consumption. Individual question scores range from zero to four. At risk alcohol consumption is indicated by a total score of five or more for males or four or more for females.

Information on paid and unpaid work is collected using modified versions of questions 34-51 of the *Australian Bureau of Statistics 2006 Census *[52] relating to jobs and work. Participants are asked to indicate whether there has been any change in employment circumstances since their diagnosis. Specific barriers to return to work are determined using the short form of the *Job Content Questionnaire *(JCQ) [53]. This widely used measure assesses job demands, control over work and support received.

*Household economic hardship *is determined by a series of questions about failure to make household payments and whether there was help provided by any organisation or individual to meet these payments. An advantage of this measure is that it is sensitive to the possibility that individuals prioritise certain payments (e.g. default on power bills to pay rent). The basis for these questions is the US Census Survey of Income and Program Participation [54].

The *Family Adaptation, Partnership, Growth, Affection, Resolve *(APGAR) [55] questionnaire is a valid and reliable 5-item instrument for assessing perceptions of adequacy of social support among adults and children. Items are rated using a three point scale from 'hardly ever' to 'almost always'. Total scores range from 0 (perceives inadequate support) to 10 (perceives adequate social support).

The *Inventory of Family Protective Factors *(IFPF) [56] is a 16-item scale which assesses stressful events the family may have experienced and how they were handled. Item scores range from 1 (not at all like my family) to 5 (almost always like my family). Total scores range from 16 to 80 with high scores indicating a perceived high degree of protection provided by the family.

The *Hospital Anxiety and Depression Scale *(HADS) [57] is used to collect information on depression and anxiety. The HADS is a self-report instrument designed for use with medically ill patients. Scores of 8 (possible range 0-21) or more on the depression or anxiety subscales are classified as 'depressed' or 'anxious', respectively. Consent has been obtained to convey scores of 8 or more directly to participants' nominated GP who will be permitted to arrange treatment or formal referral for abnormal mood symptoms according to their clinical judgement.

The *General Health Questionnaire *(GHQ-12) [58] assesses psychiatric morbidity including general level of happiness, experience of depressive and anxiety symptoms, and sleep disturbance over the last four weeks. The four-item responses range from 0 (not at all) to 1 (much more than usual) with bimodal item scoring. Total scores range from 0 to 12 with scores of 4 or more considered experiencing high psychiatric morbidity.

The *HIV Stigma Scale *[59] has been modified for use in people with epilepsy. This scale assesses 1) Personalized stigma: consequences of other people knowing they have epilepsy 2) Disclosure concerns 3) Negative self image: not as good as others, shame, guilt and 4) Public attitudes: what people think about epilepsy. The four-item responses range from 1 (strongly disagree) to 4 (strongly agree) with total scores ranging from 10 to 40 with lower scores indicating less experienced or perceived stigma.

The first seven items of the *Pittsburgh Sleep Quality Index *(PSQI) [60] are used to measure sleep quality over the previous month. Item responses range from 0 (not during the past month) to 3 (three or more times per week) with higher scores indicating more sleep disturbance.

The *Quality of Life in Epilepsy for Adolescents *(QOLIE-AD-48) [61] scale assesses the affects of epilepsy and antiepileptic medications, and daily activities for adolescents aged between 11 and 18 years of age. Items are rated on a 5 point scale from 1 (poor/much worse) to 5 (much better) with higher scores indicating better health-related quality of life.

Selected sections of questions from the *Longitudinal Study of Australian Children *(LSAC) [62] are used to assess child care, education (use, level, absence and performance) and mood.

The *Telephone Interview for Cognitive Status *(TICS-M) [63] has been validated for assessment of cognitive function for research purposes. The 13-item TICS-M test includes orientation, recent and delayed memory, attention and comprehension assessment with a maximum score of 39. As the TIICS-M can be administered via the telephone it can be used in people with visual difficulties or poor hand-eye co-ordination.

#### Diagnostic committee review

In all cases, information gathered from the treating clinician's notes through the diagnostic proforma and the baseline clinical interview with the participant will be reviewed by the Diagnostic Committee for a final decision as to the diagnosis. The diagnostic committee comprises a panel of Sydney epilepsy specialists. Epilepsy will be defined according to the ILAE guidelines [64]. A clinical diagnosis will rely upon two criteria:

(a) Clear evidence of recurrent epileptic seizures, with evidence that these are unprovoked by any acute medical condition or transient brain disorder; and

(b) Documentation of diagnosis by someone with appropriate specialized training in the recognition of epilepsy. This allows for the inclusion of those who have an enduring predisposition to generate epileptic seizures yet have only had one seizure.

Each participant will be allocated to one of the following groups:

*Definite epilepsy*, with primary documentation that meets criteria: a) clear evidence of two or more unprovoked epileptic seizures that have occurred over interval(s) exceeding 24 hours, or b) confirmed diagnosis of epilepsy by a health care provider with appropriate specialised training in the recognition and treatment of epilepsy.

*Probable epilepsy*, with other sources of information indicating the likelihood that criteria (a) or (b) are met. Documentation of a diagnosis of epilepsy by a trained non-specialist health care provider without specific documentation of definite criteria above.

*Possible epilepsy*: requires further information from the baseline interview to confirm this and identify the seizure type classification.

*Other seizure type: *provoked seizure (acute symptomatic) e.g. post acute head injury, non-epileptic non-organic seizures (panic, psychogenic etc), non-epileptic organic seizures (syncope etc)

Not epilepsy

### Statistical considerations

#### Sample size assumption

In the most recent census [52] there were 510,000 people usually resident in the Eastern zone of the SSWAHS. SEISMIC can expect to recruit 254 people with epilepsy per year for a total of 508 over the two year recruitment period. With an estimated 5% refusal, we can expect 484 people to be recruited.

##### Adults

In a Tasmanian cross sectional survey approximately 30% of adults with epilepsy reported psychological morbidity, which is associated with increased disability (e.g. taking more sickness or disability days). In an American study having chronic epilepsy and depression was associated taking 4 more disability days per month than those without [65]. If we hypothesise that participants with psychiatric co-morbidity experience 5 disability days vs. 3 disability days per month for those without psychiatric co-morbidity and the standard deviation (SD) of these is 4 then we are looking for an effect size of 0.5. With 90% power, an alpha of 0.05 would require a sample of 191 (64 with psychiatric co-morbidity, 127 without) to detect this difference in disability days per month (total sample 143 at 80% power). With the incidence assumptions from above, two years of recruitment would be adequate to detect this in working age adults and evaluate employment consequences of epilepsy as well as providing some leeway for multivariate modelling and interactions.

##### Children

In the SSWAHS 39% of families speak a language other than English at home. If we hypothesis that this is associated with a greater economic household impact through e.g. increased likelihood of not making a basic household payment, then the sample size recruited in two years above is sufficient to detect a relative risk of 1.5 for this poorer socioeconomic outcome.

### Statistical Analysis

The incidence of epilepsy will be reported as definite cases of epilepsy over the population of the study area. Incidence will also be reported by age and syndromic groups. Data analysis will use multivariable one-level (SAS Proc Genmod) as well as mixed model approaches, which take variations within cluster (patients) and between clusters (e.g. hospitals) into account (SAS Proc Mixed). Mixed model approaches will be of use when cluster and individual level data are used to explain a possible outcome, for example, process of care by hospital along with individual level data on language spoken at home as risk factors for adherence to medication.

Multivariable regression will be used to examine the contributions of protective and risk factors on several clinical, psychosocial and economic outcomes. Hypothesised and known risk factors from the literature will be used in the initial regression models, together with factors with a p-value of less than 0.2 in univariate analyses which will be considered as potential confounders. Relative risks and their corresponding 95% confidence intervals will be presented for risk factors as they relate to the outcomes of interest.

Since the length of follow-up for participants will differ depending on the date of diagnosis and hence enrolment into the incident cohort, length of time in the study (and therefore at risk) will be taken into account in regression models. Potential effect modifiers will be examined by inserting interaction terms into the multivariable models.

Survival analyses will be used to investigate the time trajectory of risk and protective factors for various poor outcomes. Kaplan-Meier survival curves will be produced and then the adjusted survival will be examined using Cox proportional hazards models in order to take into account multiple exposure variables.

### Ethical approval

Full ethical approval was provided by the Human Research Ethics Committee/08/RPAH/258 (lead committee) of the SSWAHS for protocol No X08-0152 on 10^th ^July 2008 and from local institutional research governance offices for each clinical centre or PI. Written informed consent is obtained for every participant or their parent, carer, guardian or proxy.

## Discussion

Epilepsy is a costly, stigmatized and neglected condition. With the rapidly changing demographic and ethnic makeup of the Australian population, a population-based study of this condition is timely. Through SEISMIC the rates of different epilepsy syndromes, overall and by sex and age group, will be determined for Australia. A feature of this study is its multidisciplinary approach in assessing the impact of epilepsy on individuals, their families and to the community.

SEISMIC will contribute information to promote a healthy life by providing information that will help address the psychosocial and economic impact associated with epilepsy which will ultimately help people live fulfilling and productive lives. The current paucity of prospectively collected population-based Australian data on people with epilepsy hinders the development of programs to reduce this burden. For the first time, data on psychosocial well-being, economic hardship, stigma, and service use following epilepsy will be collected.

## Competing interests

The authors declare that they have no competing interests.

## Authors' contributions

CA is the principal investigator of SEISMIC and is responsible for the academic oversight of the study and the integrity of the data. MH drafted this manuscript which is based on the original SEISMIC study protocol drafted by NG, MH and AM. CA, NG, MH and AM jointly developed and wrote the protocol from its inception. SJ designed the economic analyses. AM designed the stigma component of the study. All authors were involved in revising the manuscript and gave final approval for publication.

## The SEISMIC collaborative group*

### Advisory Committee

Craig Anderson, Sam Berkovic (C), Wendyl D'Souza, John Dunne, Michelle Kiley, Cecilie Lander, Sue Ronaldson

#### Grant Holders

Craig Anderson (PI), Andrew Bleasel, Nick Glozier, Maree Hackett, Stephen Jan, John Lawson, Alexandra Martiniuk, Armin Mohammad, Ernie Somerville

#### Operational Group

Craig Anderson, Lucy Arblaster, Beverley Essue, Maree Hackett (C), Lorne Hyde, Stephen Jan, Lisa Todd

#### Steering Committee

Craig Anderson (C), Lucy Arblaster, Andrew Bleasel, Alastair Corbett, Nick Glozier, Gillian Gardner, Daniel Ghougassian, Deepak Gill, Maree Hackett, Bassel Hassan, Carol Ireland, Stephen Jan, John Lawson, Alexandra Martiniuk, Armin Mohammad, Maryanne Ng, Ernie Somerville, Lisa Todd

#### Clinical Investigators

Craig Anderson, Michael Barnett, Andrew Bleasel, Bruce Brew, Peter Brimage, Alastair Corbett, Lyndal Douglas, Daniel Ghougassin, Deepak Gill, Bassel Hassan, Natalie Hitchens, John Lawson, Joanne Leal, Armin Mohammad, Jenine Murray, Hyunmin Park, Hugo Alexandre Sampaio, David Sharpe, Ernie Somerville, Penny Spring, Con Yiannikas

PI = Principal investigator

C = Chairperson

#### Project managers

Ms Lucy Arblaster (current), Ms Maryanne Ng (on leave), Mr Dan Jackson (previous), Ms Carol Burke (previous).

*Correct at the time of publication

## Pre-publication history

The pre-publication history for this paper can be accessed here:

http://www.biomedcentral.com/1471-2377/11/3/prepub
